# Index to Original Communications in Medical Journals of the United States and Canada

**Published:** 1879-04

**Authors:** 


*Index to Original Communications in the Medical Journals of the United States and Canada, for* 1877. Classified by subjects and authors. Compiled by WM. D. CHAPIN. New York. Price One Dollar.

All authors who are investigating special subjects must be most deeply interested in this little volume. The valuable original articles in all American and Canadian Journals may through the aid of this index be easily referred to, thus adding to the value of the journals and greatly facilitating reference. We regard it as *invaluable*, and believe this will be the unanimous verdict of the profession. Index for 1878 is being compiled by the same author. National Printing Company, 16 Chambers Street, New York.

